# Supplementary material for: Karyotypic Flexibility of the Complex Cancer Genome and the Role of Polyploidization in Maintenance of Structural Integrity of Cancer Chromosomes
Source: Cancers (Basel). 2020 Mar 5;12(3):591. doi: 10.3390/cancers12030591 (PMC7139464; doi:10.3390/cancers12030591)
Supplement: Supplementary file 1 [file cancers-12-00591-s001.pdf]

cancers

MDPI

Article

Karyotypic Flexibility of the Complex Cancer Genome and the Role of Polyploidization in Maintenance of Structural Integrity of Cancer Chromosomes

Christina Raftopoulou, Fani-Marlen Roumelioti, Eleni Dragona, Stefanie Gimelli, Frédérique Sloan-Béna, Vasilis Gorgoulis, Stylianos E. Antonarakis and Sarantis Gagos

Supplementary Materials

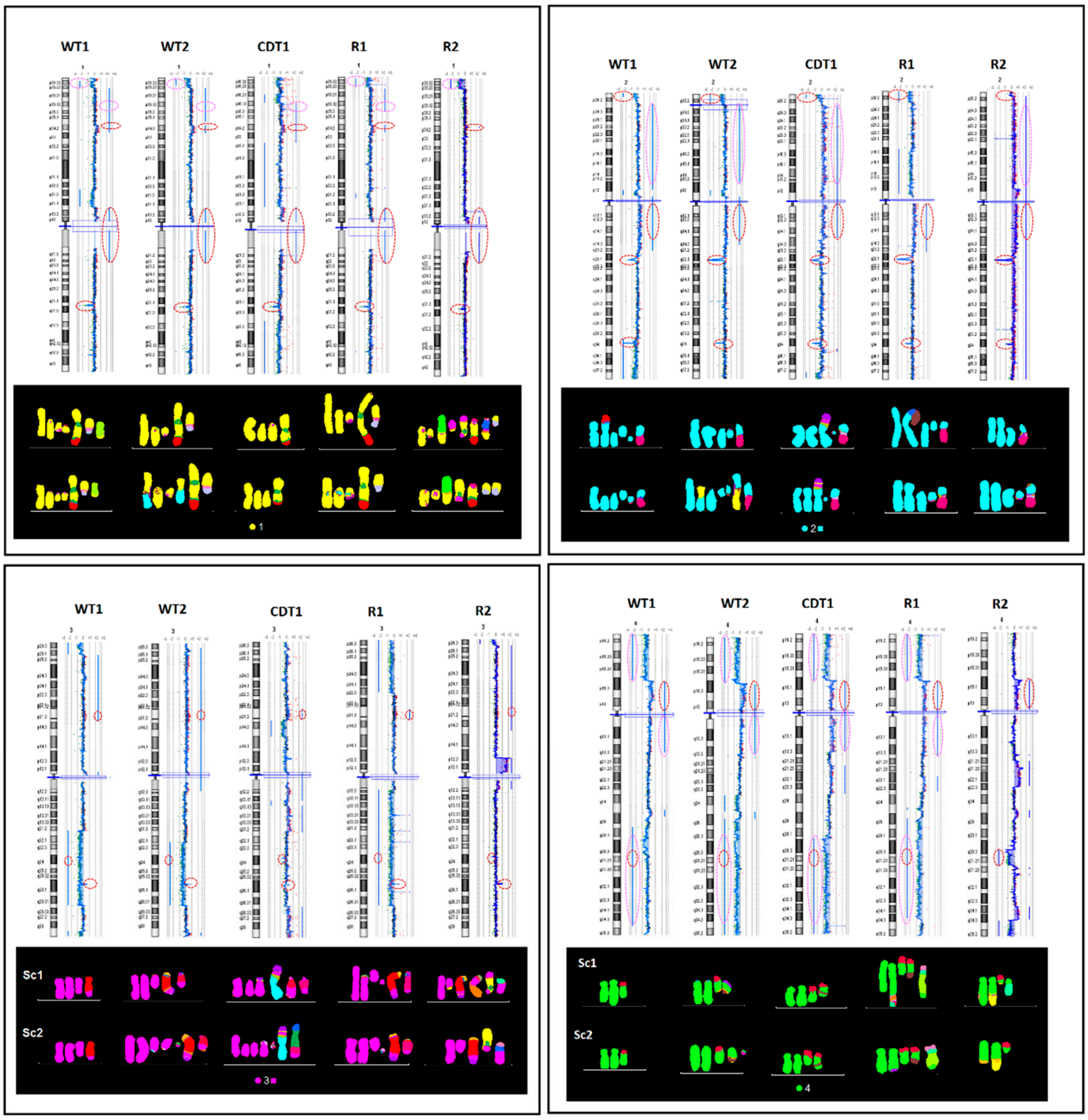

Figure S1. Comparison of aCGH with M-FISH in 5 U2-OS cell lines. Human chromosomes 1–4: Identical large genome imbalances identified by aCGH, are stably maintained between the U2-OS derivative cell lines despite the karyotypic alterations produced by extensive chromosome breakage and illegitimate rejoining (red circles indicate presence of the imbalance in all 5, whereas pink circles depict undistinguishable duplications/deletions in 4 out of 5 karyotypically diverse U2-OS cell lines.

Black boxes include partial molecular karyotypes from two major subclones from each of the 5 U2-OS cell lines, representing the above arrayed chromosomes.

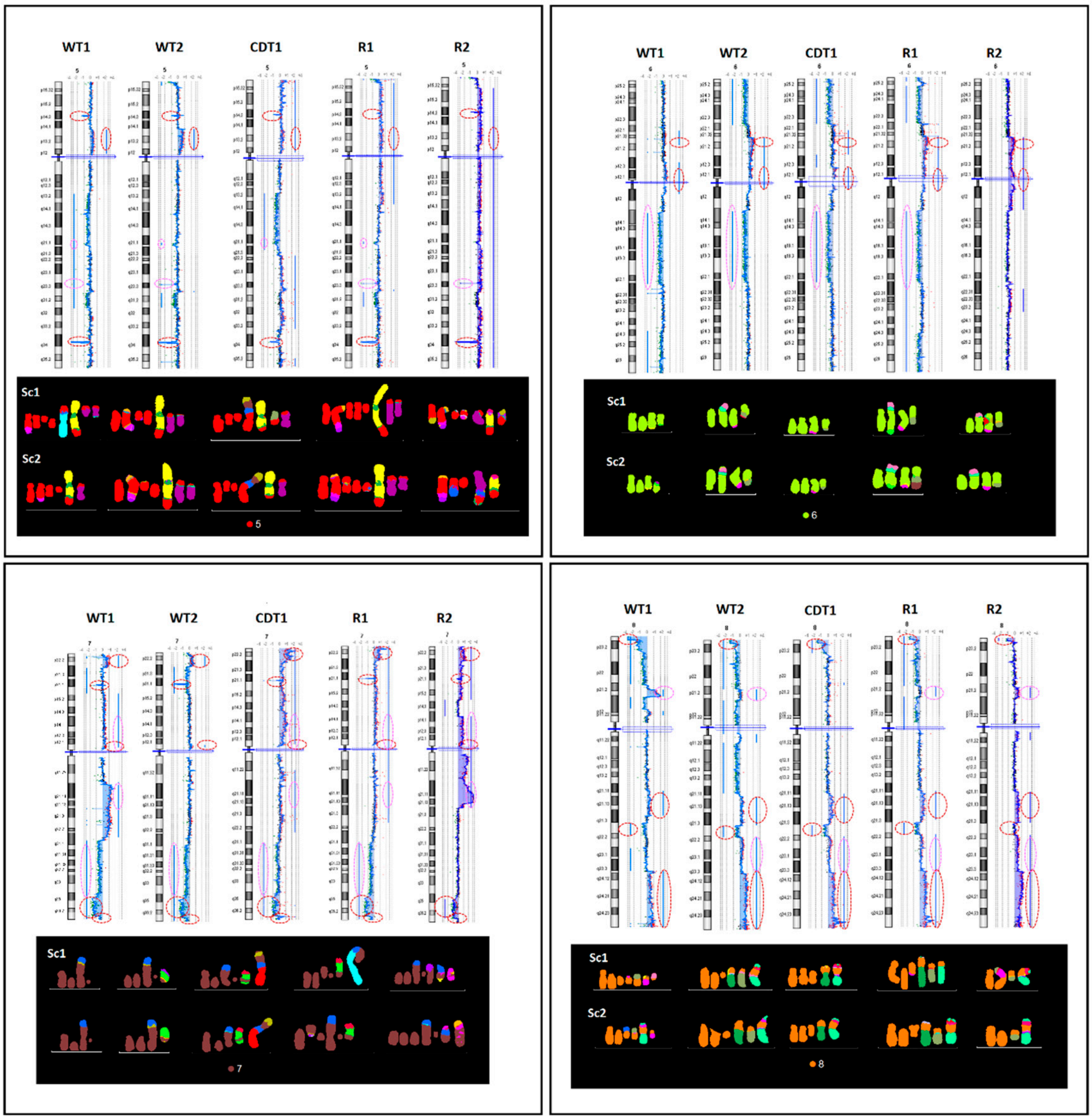

Figure S2. Comparison of aCGH with M-FISH in 5 U2-OS cell lines. Human chromosomes 5–8. Identical large genome imbalances identified by aCGH, are stably maintained between the U2-OS derivative cell lines despite the karyotypic alterations produced by extensive chromosome breakage and illegitimate rejoining (red circles indicate presence of the imbalance in all 5, whereas pink circles depict undistinguishable duplications/deletions in 4 out of 5 karyotypically diverse U2-OS cell lines. Black boxes include partial molecular karyotypes from two major subclones from each of the 5 U2-OS cell lines, representing the above arrayed chromosomes.

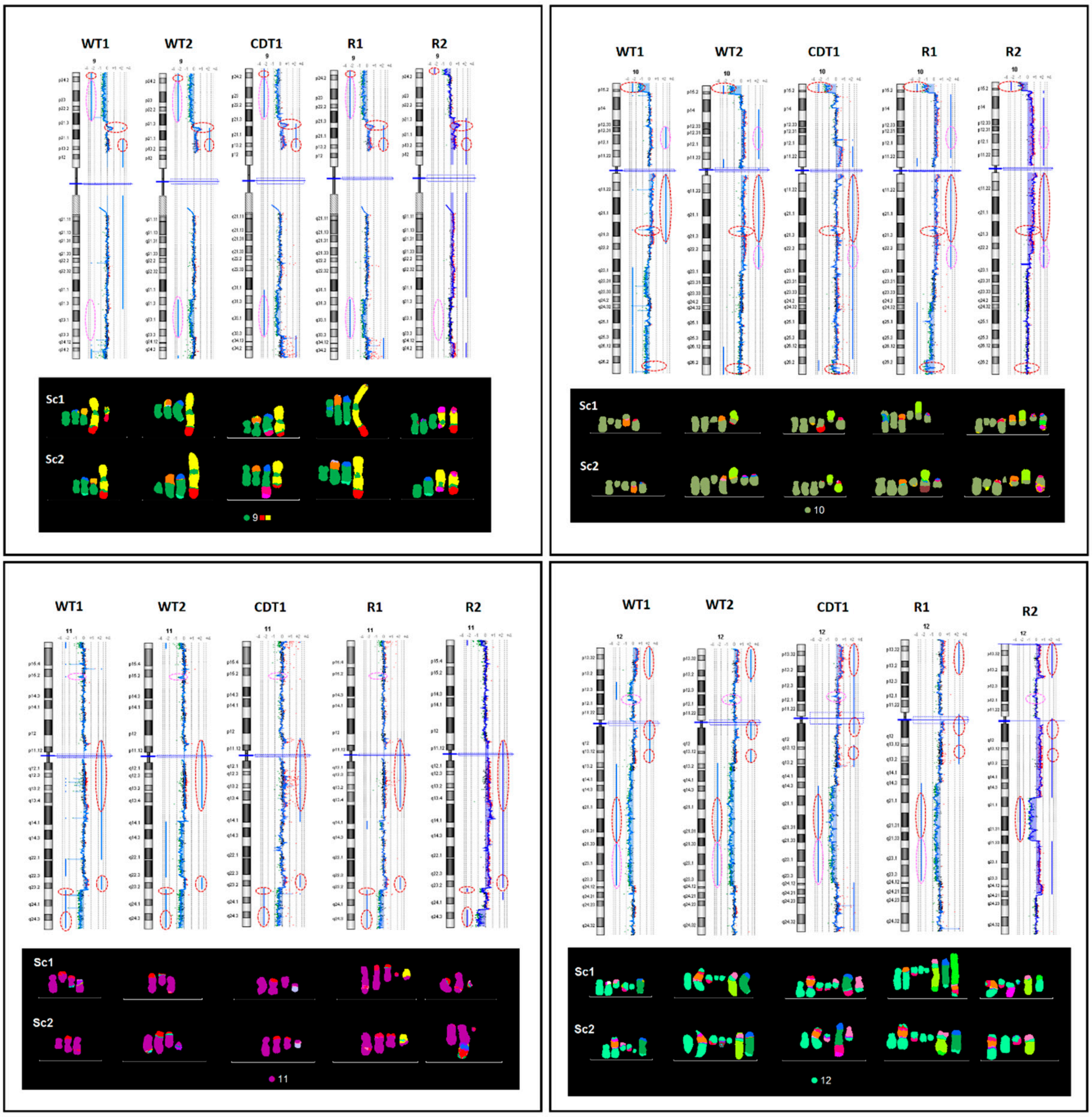

Figure S3. Comparison of aCGH with M-FISH in 5 U2-OS cell lines. Human chromosomes 6–12. Identical large genome imbalances identified by aCGH, are stably maintained between the U2-OS derivative cell lines despite the karyotypic alterations produced by extensive chromosome breakage and illegitimate rejoining (red circles indicate presence of the imbalance in all 5, whereas pink circles depict undistinguishable duplications/deletions in 4 out of 5 karyotypically diverse U2-OS cell lines. Black boxes include partial molecular karyotypes from two major subclones from each of the 5 U2-OS cell lines, representing the above arrayed chromosomes.

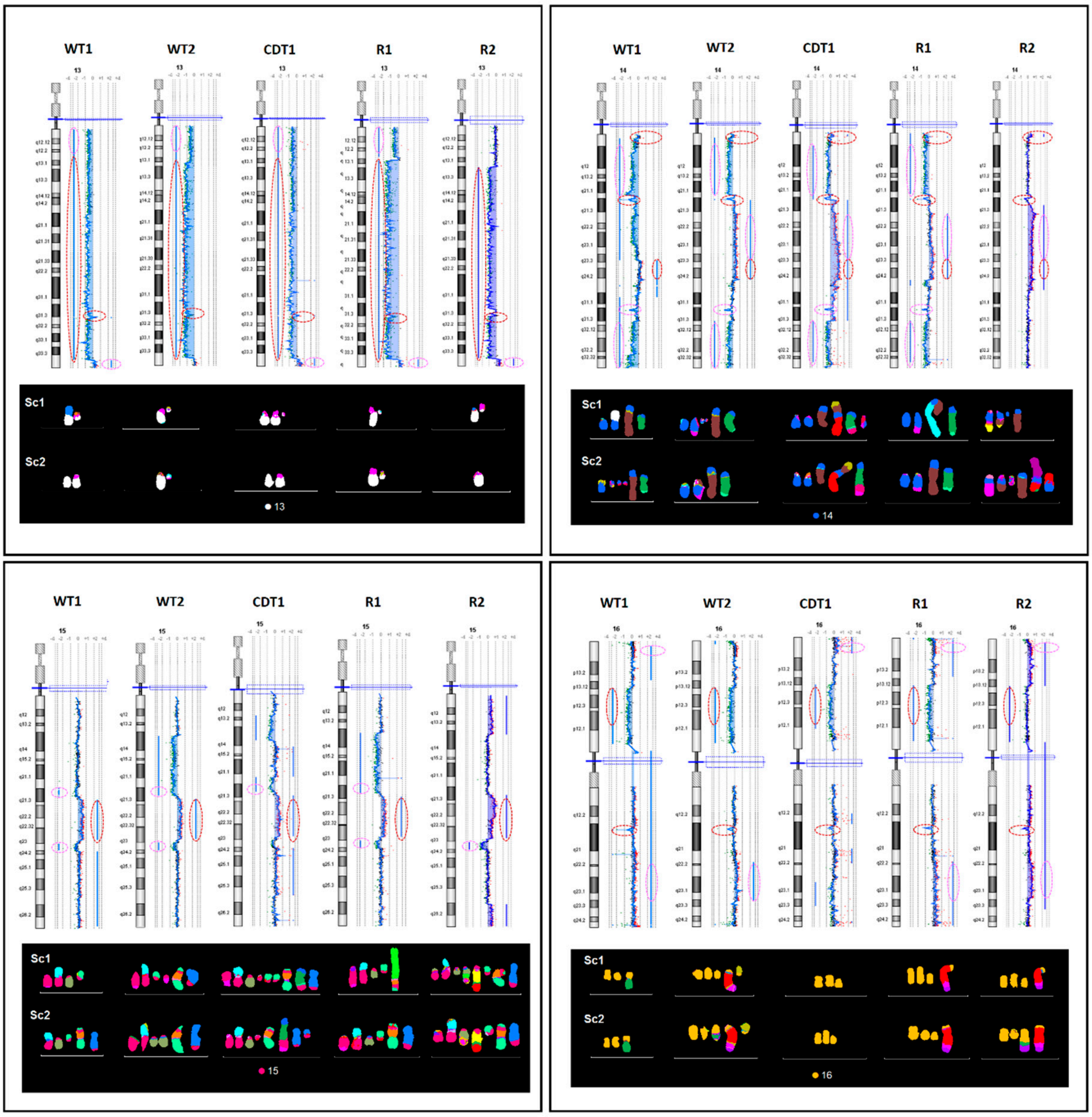

Figure S4. Comparison of aCGH with M-FISH in 5 U2-OS cell lines. Human chromosomes 13-16. Identical large genome imbalances identified by aCGH, are stably maintained between the U2-OS derivative cell lines despite the karyotypic alterations produced by extensive chromosome breakage and illegitimate rejoining (red circles indicate presence of the imbalance in all 5, whereas pink circles depict undistinguishable duplications/deletions in 4 out of 5 karyotypically diverse U2-OS cell lines. Black boxes include partial molecular karyotypes from two major subclones from each of the 5 U2-OS cell lines, representing the above arrayed chromosomes.

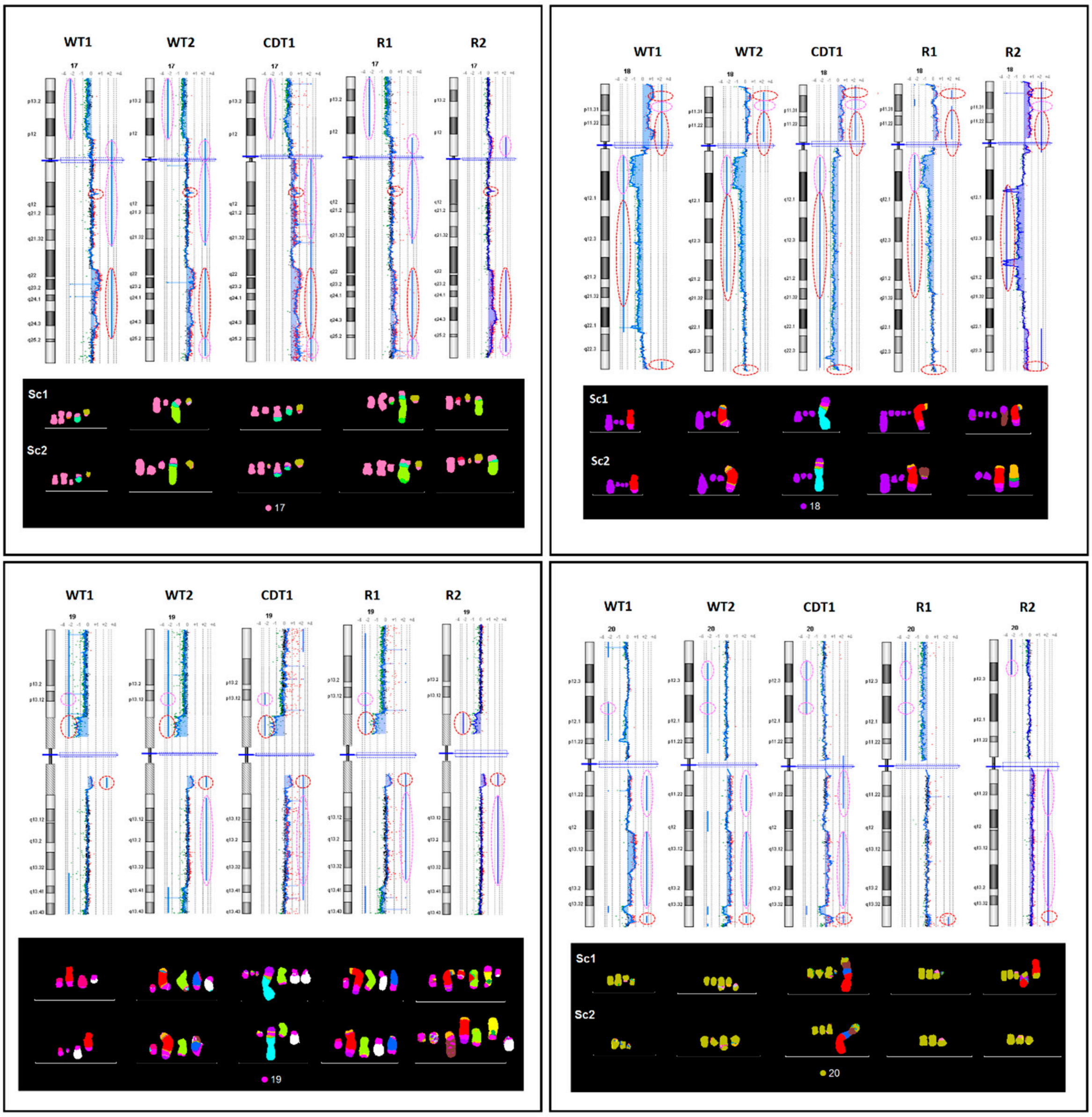

Figure S5. Comparison of aCGH with M-FISH in 5 U2-OS cell lines. Human chromosomes 17-20. Identical large genome imbalances identified by aCGH, are stably maintained between the U2-OS derivative cell lines despite the karyotypic alterations produced by extensive chromosome breakage and illegitimate rejoining (red circles indicate presence of the imbalance in all 5, whereas pink circles depict similar duplications/deletions in 4 out of 5 karyotypically diverse U2-OS cell lines. Black boxes include partial molecular karyotypes from two major subclones from each of the 5 U2-OS cell lines, representing the above arrayed chromosomes.

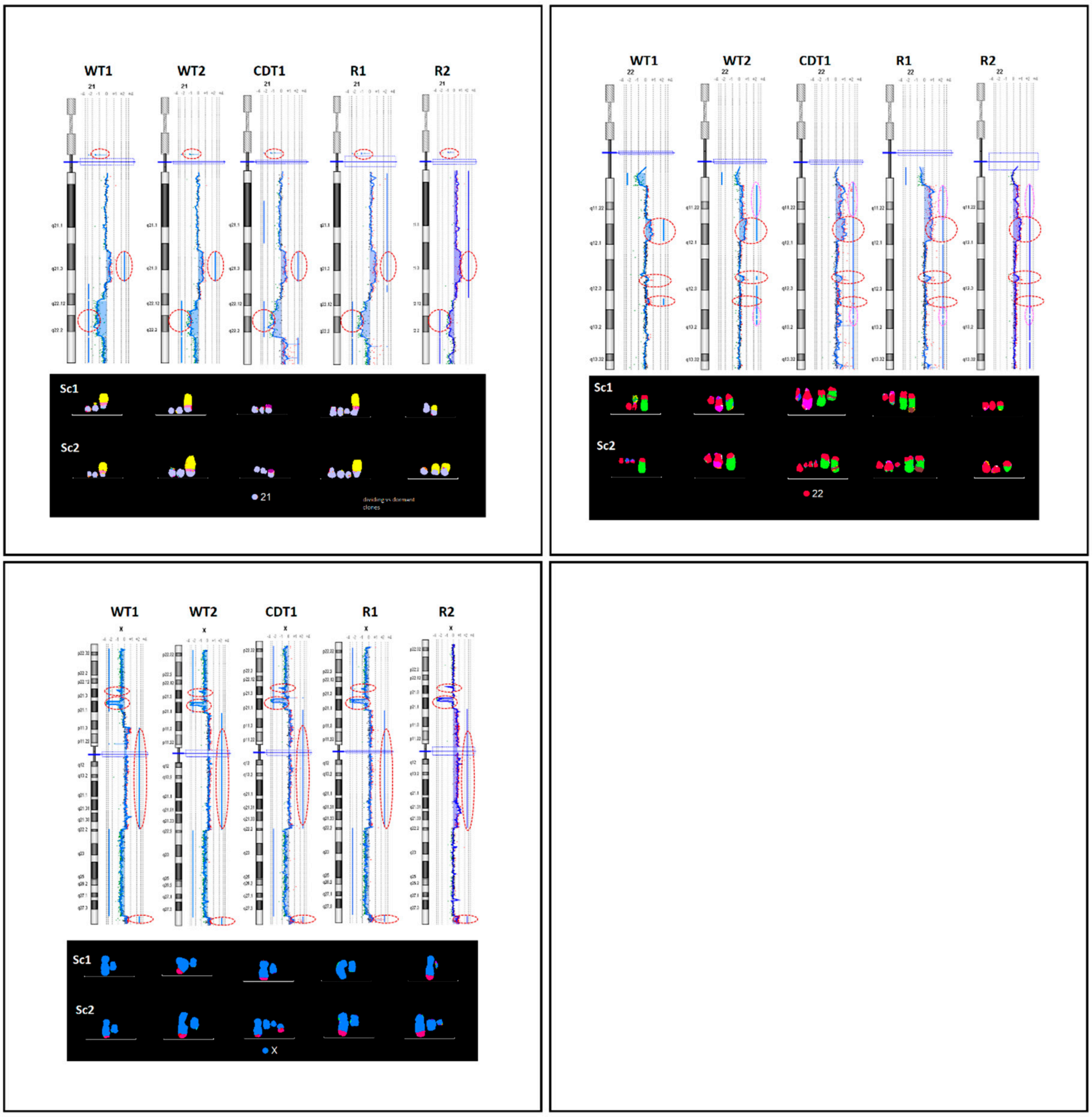

Figure S6. Comparison of aCGH with M-FISH in 5 U2-OS cell lines. Human chromosomes 21-22 and X. Identical large genome imbalances identified by aCGH, are stably maintained between the U2-OS derivative cell lines despite the karyotypic alterations produced by extensive chromosome breakage and illegitimate rejoining (red circles indicate presence of the imbalance in all 5, whereas pink circles depict undistinguishable duplications/deletions in 4 out of 5 karyotypically diverse U2-OS cell lines. Black boxes include partial molecular karyotypes from two major subclones from each of the 5 U2-OS cell lines, representing the above arrayed chromosomes. The U2-OS cells derive from a female patient [Ponten and Saksela, 1967].

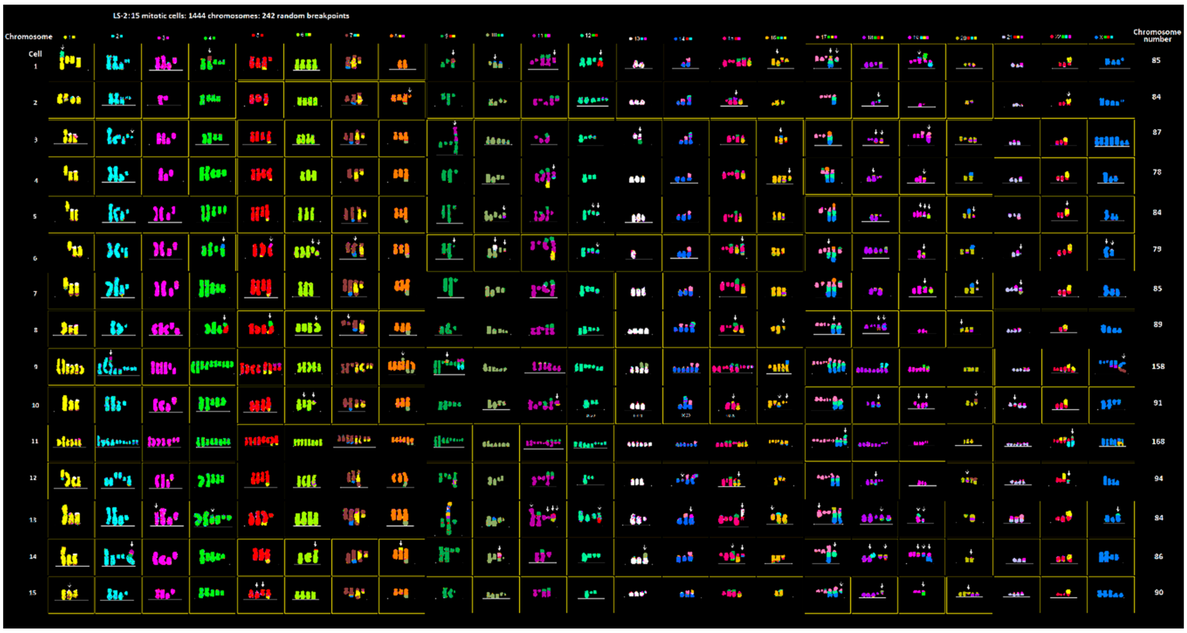

Figure S7. Random chromosome rearrangements in 15 LS-2 cells. Pseudo-colored molecular karyograms from 15 randomly picked, co-dividing LS-2 cells. Chromosome number per metaphase is displayed. Arrows indicate random non-clonal structural rearrangements. Cells 9 and 11, underwent WGD (242 random breakpoints in 1444 chromosomes).

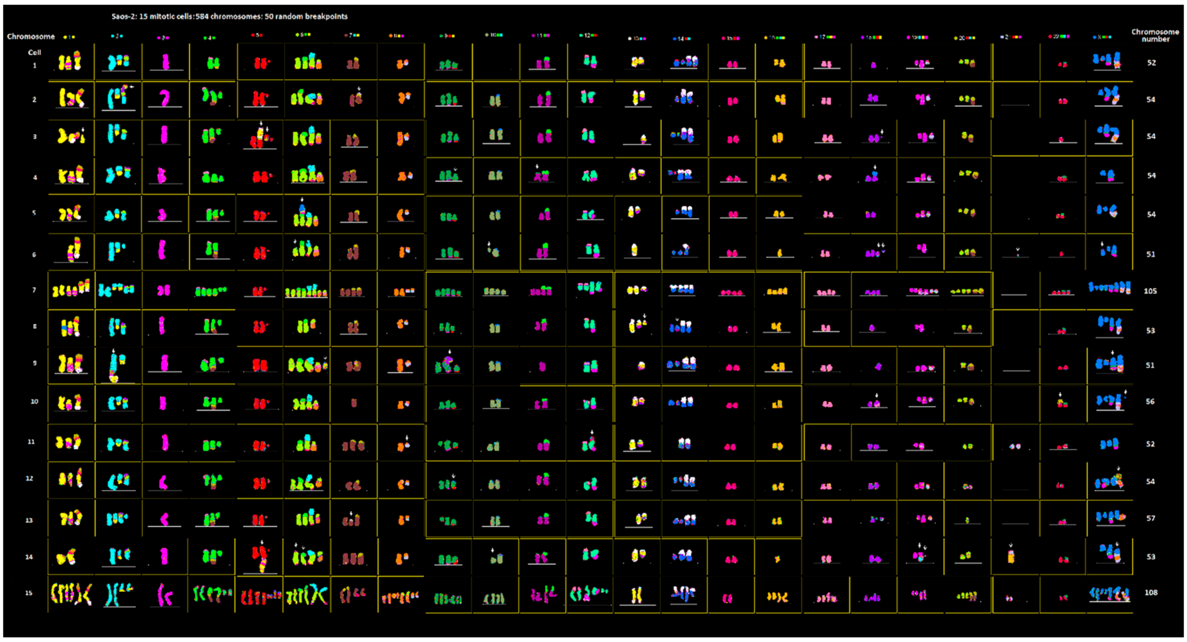

Figure S8. Random chromosome rearrangements in 15 Saos-2 cells. Pseudo-colored molecular karyograms from 15 randomly picked co-dividing Saos-2 cells. Chromosome number per metaphase is displayed. Arrows indicate random non-clonal structural rearrangements. Cells 7 and 15, underwent WGD (50 random breakpoints in 584 chromosomes).

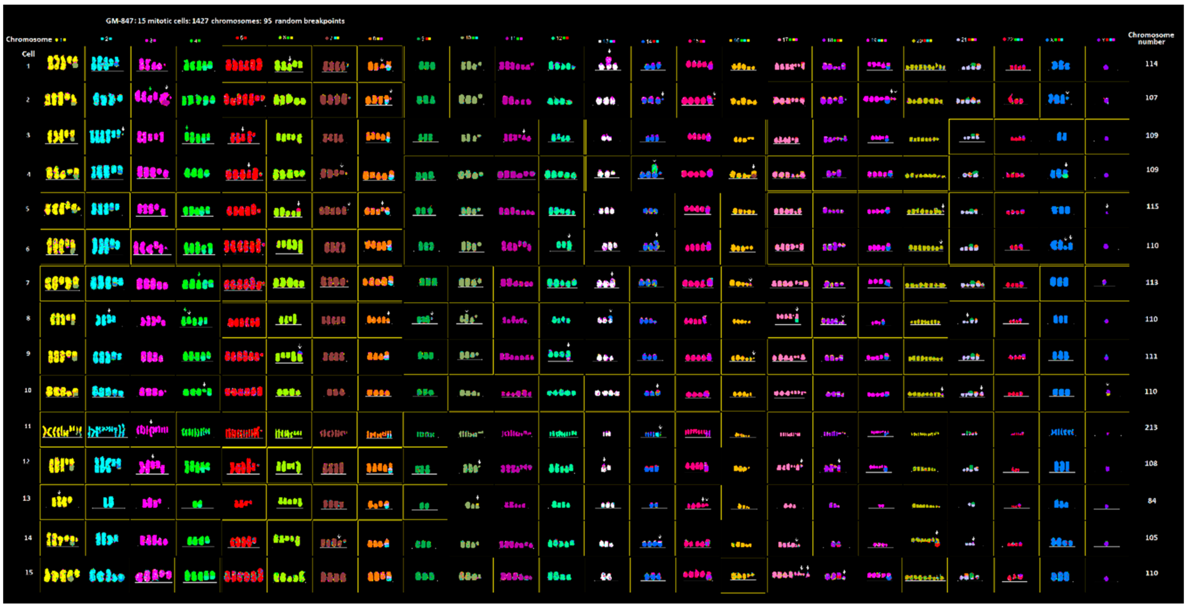

Figure S9. Random chromosome rearrangements in GM-847-2 cells. Pseudo-colored molecular karyograms from 15 randomly picked co-dividing GM-847 cells. Chromosome number per metaphase is displayed. Arrows indicate random non-clonal structural rearrangements. Cell 11 underwent WGD (95 random breakpoints in 1427 chromosomes).

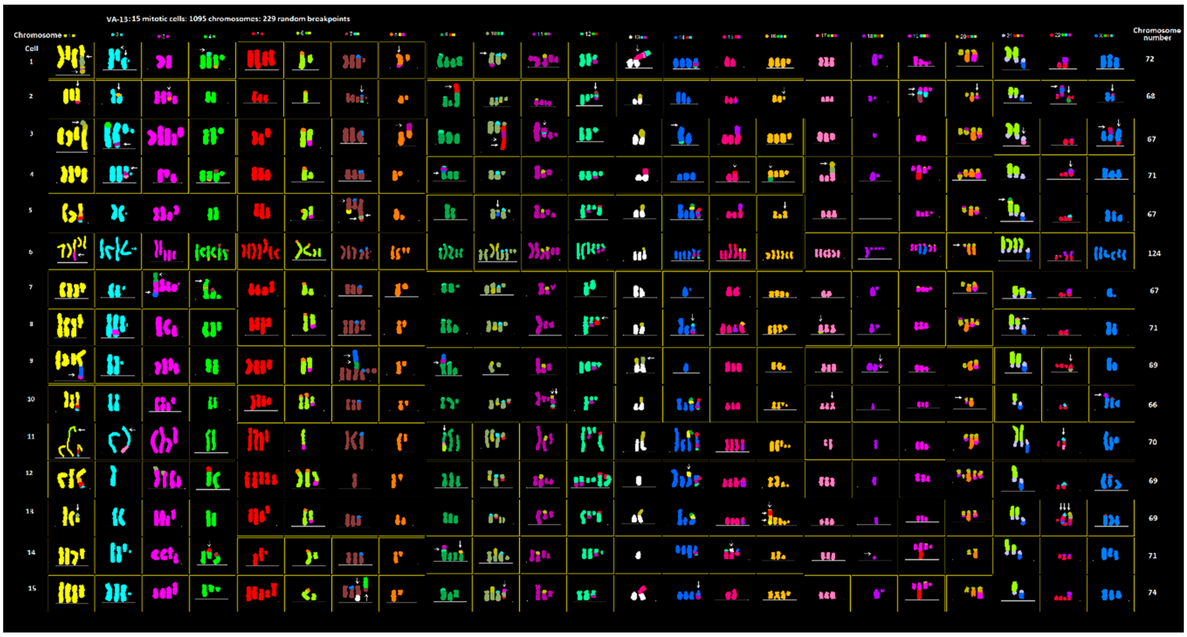

Figure S10. Random chromosome rearrangements in VA-13 cells. Pseudo-colored molecular karyograms from 15 randomly picked co-dividing VA-13 cells. Chromosome number per metaphase is displayed. Arrows indicate random non-clonal structural rearrangements. Cell 6 underwent WGD (50 random breakpoints in 584 chromosomes). This is a meta-analysis of the dataset included in Sakelariou et al 2013.

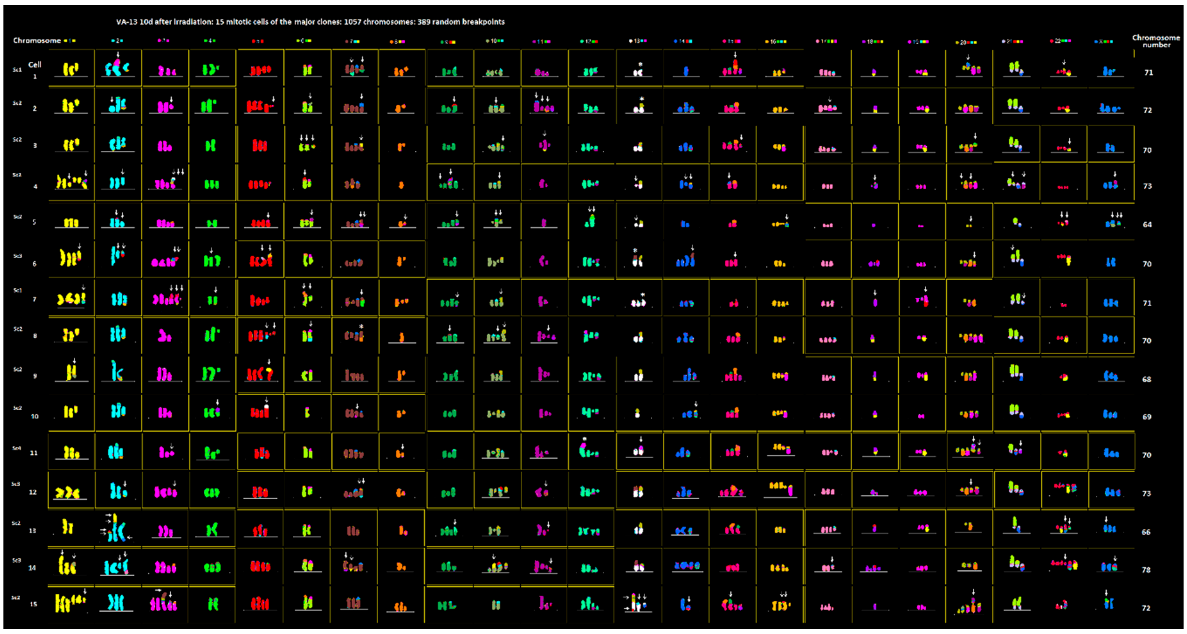

Figure S11. Random chromosome rearrangements in irradiated VA-13 cells-major sub-clones. Pseudo-colored molecular karyograms from 15 randomly picked co-dividing VA-13 cells representing the major numerical chromosomal constitution of 64–78 chromosomes, 10 days post 2.4 Gy of gamma-irradiation. Chromosome number per metaphase is displayed. Arrows indicate random non-clonal structural rearrangements. SC1-3 indicate cells belonging in karyotypically discrete sub-clones. Asterisks indicate sub-clone-specific structural aberrations of chromosome 13 (389 random breakpoints in 1057 chromosomes).

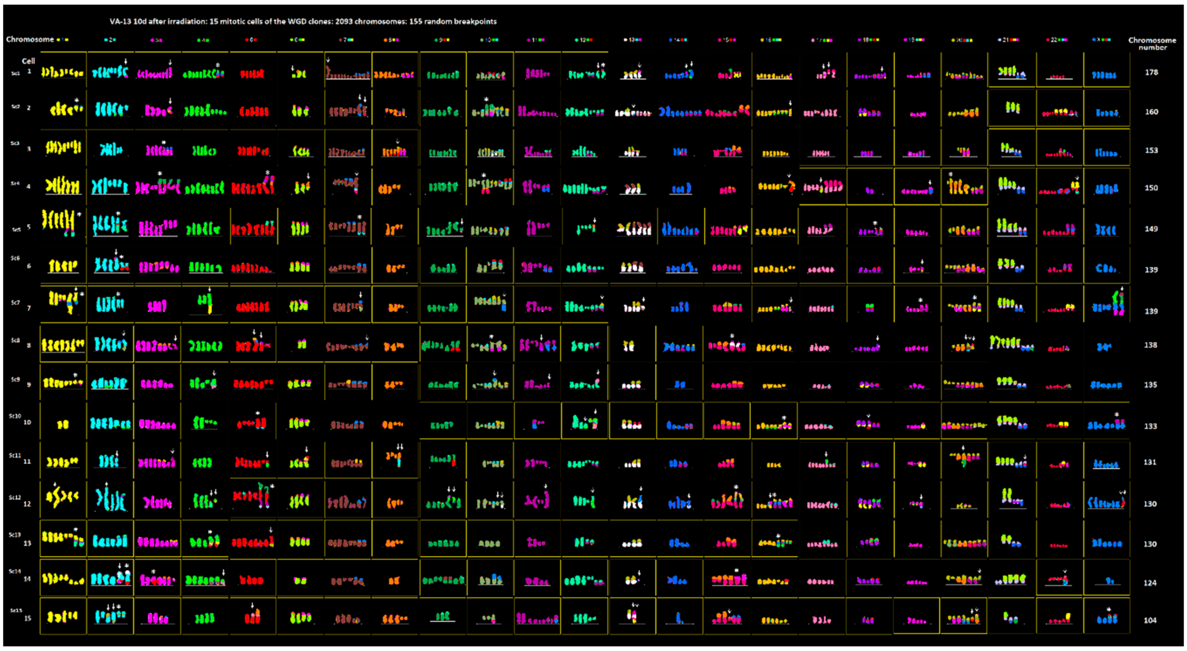

Figure S12. Random chromosome rearrangements in irradiated VA-13 cells-WGD sub-clones. Pseudo-colored molecular karyograms from 15 randomly picked co-dividing VA-13 cells with endoreduplicated chromosomal constitution of 104–178 chromosomes, 10 days post 2.4 Gy of gamma-irradiation. Chromosome number per metaphase is displayed. Arrows indicate random non-clonal structural rearrangements. According to our criteria for clonality of rearrangements, all 15 cells of this group derived from at least two divisions, the first occuring in the non-endoreduplicated progenitor cells (Sc1-15 indicate discrete sub-clones, asterisks indicate sub-clone-specific structural aberrations) (155 random breakpoints in 2093 chromosomes).

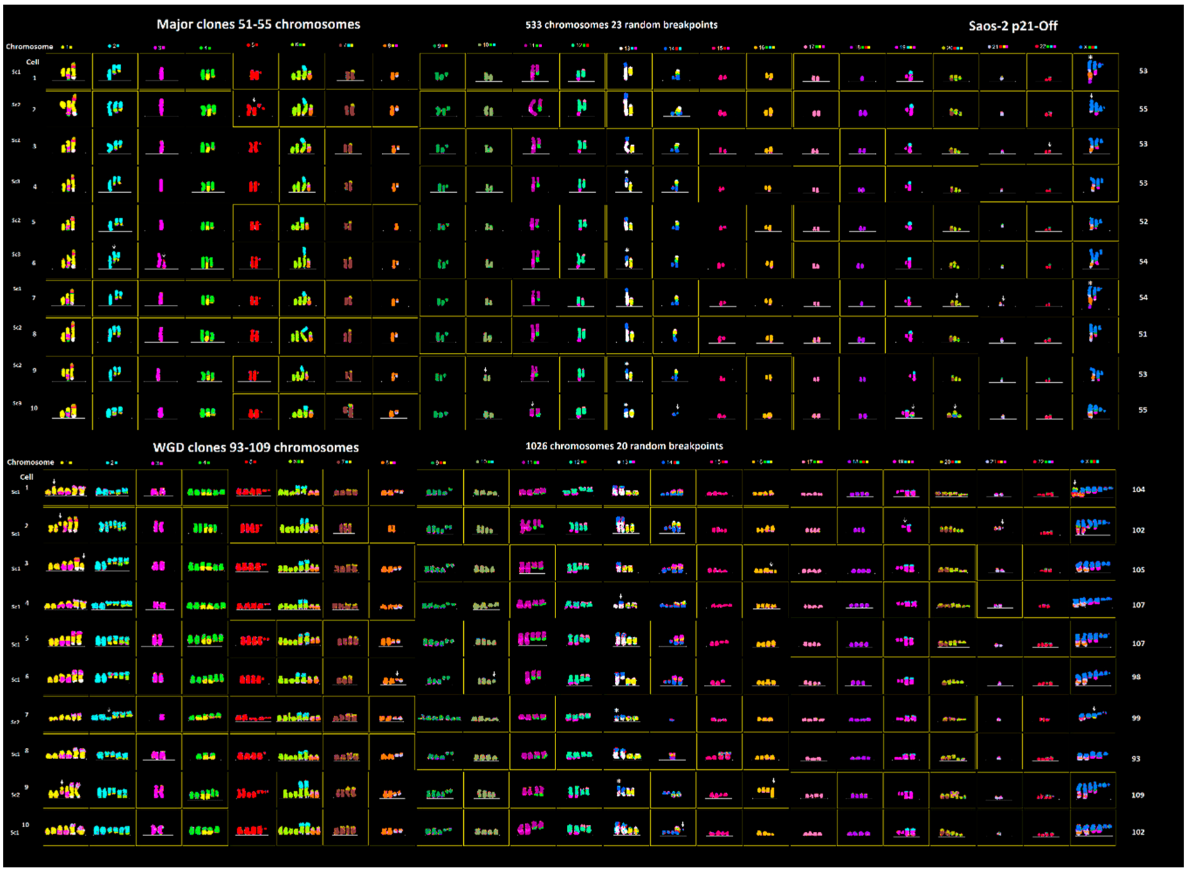

Figure S13. Random chromosome rearrangements in control Saos2 cells. Pseudo-colored molecular karyograms from 20 randomly picked, co-dividing Saos2 p21$^{WAF1/Cip1}$ Tet-ON (control) cells not-induced for p21 overexpression. Mitotic cells are divided into two groups: 10 cells representing the major numerical chromosomal constitution of 51–55 chromosomes (upper panel) (23 random breakpoints in 533 chromosomes) and 10 cells with endoreduplicated genomic content and 93–109 chromosomes (20 random breakpoints in 1026 chromosomes). Chromosome number per metaphase is displayed. Arrows indicate random non-clonal structural rearrangements (Sc1–Sc3 indicate karyotypically distinct subclones, based on the constitution of chromosomes X and 13).

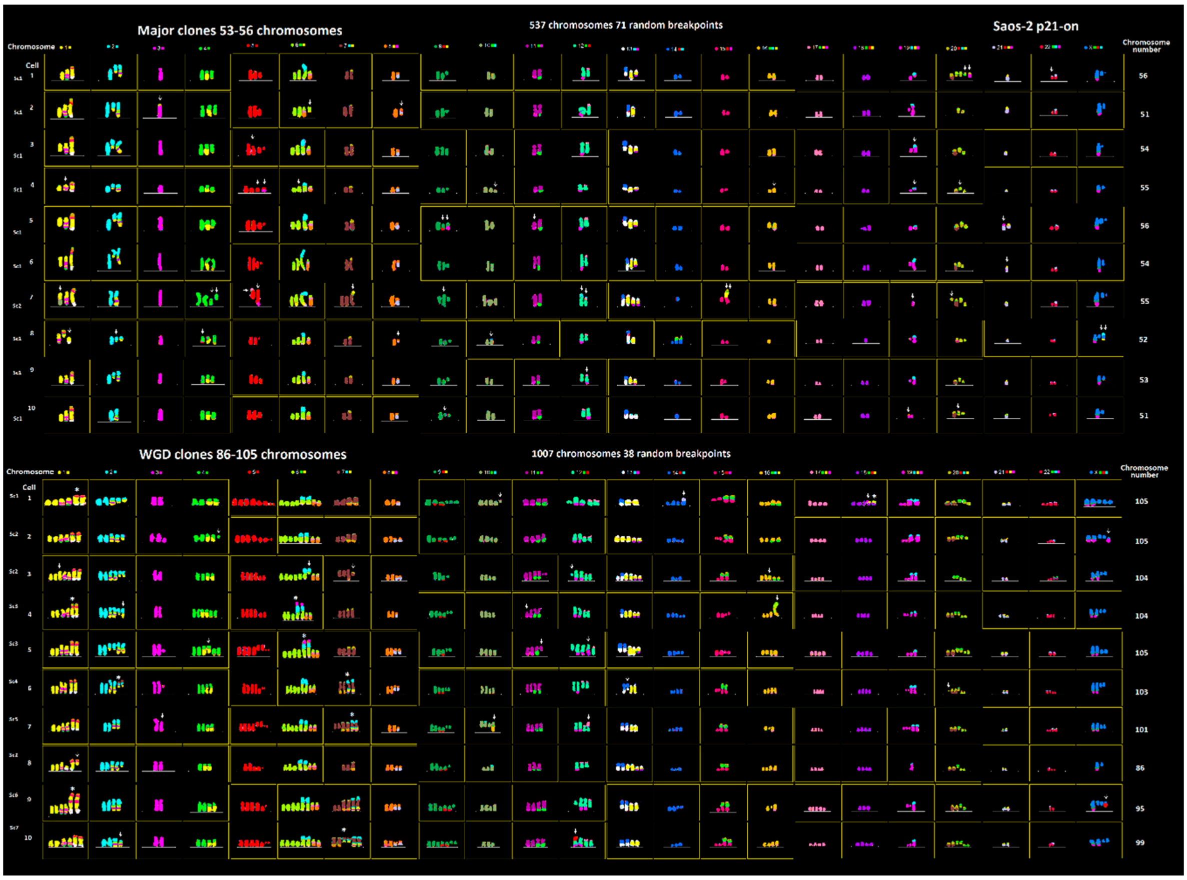

Figure S14. Random chromosome rearrangements in survivors of p21 overexpression. Pseudo-colored molecular karyograms from 20 randomly picked, co-dividing Saos2 p21$^{WAF1/Cip1}$ Tet-ON survivor cells upon p21 overexpression. Mitotic cells are divided into two groups: 10 karyograms representing the major numerical chromosomal constitution of 51-56 chromosomes (upper panel) (71 random breakpoints in 537 chromosomes) and 10, with endoreduplicated genomic content and 86–105 chromosomes (38 random breakpoints in 1007 chromosomes). Chromosome number per metaphase is displayed. Arrows indicate random non-clonal structural rearrangements. Karyotypic heterogeneity is found increased in the group of endoreduplicated cells. Sc1–Sc7 indicate karyotypically distinct subclones). Asterisks indicate duplicate copies of newly emerged structural clonal aberrations not observed in the non-endoreduplicated group of cells representing the major population.

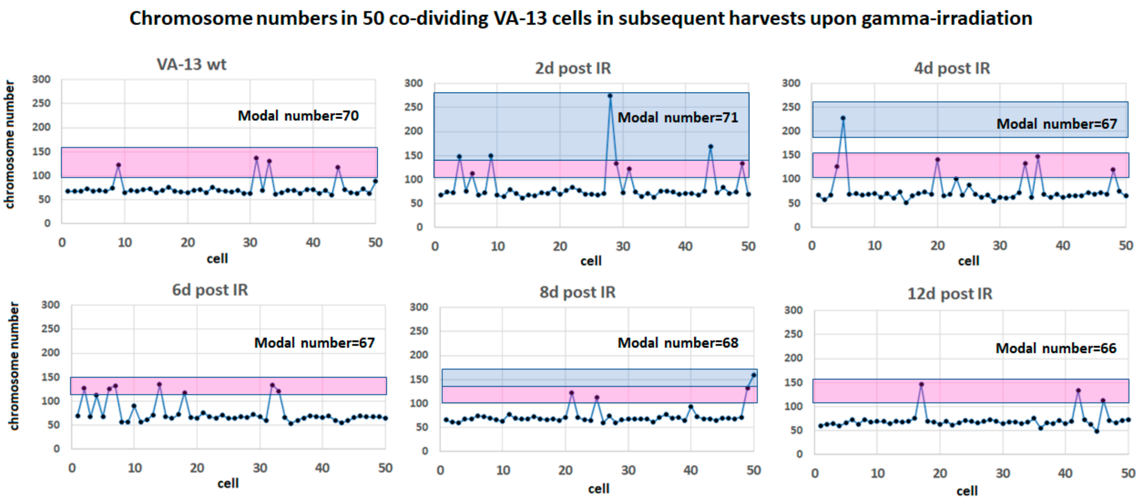

Figure S15. Chromosome numbers in 50 VA-13 cells after irradiation. Distribution of chromosome counts in 50 co-dividing VA-13 cells cultured for 12 days after 2.4 Gy of gamma irradiation. Modal chromosome number is indicated. Pink boxes include cells undergone one round of WGD. Blue boxes include cells undergone more than one round of WGD. Note that despite the decline in the rates of WGD by time in culture, very few intermediate chromosome counts were recorded between the distinct ploidy indices suggesting that cells with heavily imbalanced DNA content, are less proficient to divide.

© 2020 by the authors. Submitted for possible open access publication under the terms and conditions of the Creative Commons Attribution (CC BY) license (http://creativecommons.org/licenses/by/4.0/).
